# IQ changes after pediatric epilepsy surgery: a systematic review and meta-analysis

**DOI:** 10.1007/s00415-023-12002-8

**Published:** 2023-09-28

**Authors:** Tristan Schmidlechner, Malin Zaddach, Florian Heinen, Sonia Cornell, Georgia Ramantani, Jan Rémi, Christian Vollmar, Mathias Kunz, Ingo Borggraefe

**Affiliations:** 1 Division of Pediatric Neurology, Developmental Medicine and Social Pediatrics, Department of Pediatrics, Dr. Von Hauner Children’s Hospital, University Hospital, Ludwig-Maximilians-University Munich, Lindwurmstreet 4, 80337 Munich, Germany; 2 Department of Neuropediatrics, University Children’s Hospital, Zurich, Switzerland; 3 University of Zurich, Zurich, Switzerland; 4 Department of Neurology, University Hospital, Ludwig-Maximilians-University Munich, Munich, Germany; 5 Department of Neurosurgery, University Hospital, Ludwig-Maximilians-University Munich, Munich, Germany; 6 Comprehensive Epilepsy Center, University Hospital, Ludwig-Maximilians-University Munich, Munich, Germany

**Keywords:** Epilepsy surgery, Cognition, Children, Outcome, Neuropsychology

## Abstract

**Objective:**

This systematic review aimed to assess the intellectual outcome of children who underwent surgery for epilepsy.

**Methods:**

A systematic review of electronic databases was conducted on December 3, 2021, for PubMed and January 11, 2022, for Web of Science. The review was conducted according to the PRISMA guidelines. The included studies reported on intelligence quotient (IQ) or developmental quotient (DQ) before and after epilepsy surgery in children. Studies were included, if the patients had medically intractable epilepsy and if the study reported mainly on curative surgical procedures. We conducted a random-effects meta-analysis to determine the mean change of IQ/DQ.

**Results:**

Fifty-seven studies reporting on a total of 2593 patients met the inclusion criteria. The mean age at surgery was 9.2 years (± 3.44; range 2.4 months–19.81 years). Thirty-eight studies showed IQ/DQ improvement on a group level, 8 yielded stable IQ/DQ, and 19 showed deterioration. Pooled analysis revealed a significant mean gain in FSIQ of + 2.52 FSIQ points (95% CI 1.12–3.91). The pooled mean difference in DQ was + 1.47 (95% CI − 6.5 to 9.5). The pooled mean difference in IQ/DQ was 0.73 (95% CI − 4.8 to 6.2). Mean FSIQ gain was significantly higher in patients who reached seizure freedom (+ 5.58 ± 8.27) than in patients who did not (+ 0.23 ± 5.65). It was also significantly higher in patients who stopped ASM after surgery (+ 6.37 ± 3.80) than in patients who did not (+ 2.01 ± 2.41). Controlled studies showed a better outcome in the surgery group compared to the non-surgery group. There was no correlation between FSIQ change and age at surgery, epilepsy duration to surgery, and preoperative FSIQ.

**Significance:**

The present review indicates that there is a mean gain in FSIQ and DQ in children with medically intractable epilepsy after surgery. The mean gain of 2.52 FSIQ points reflects more likely sustainability of intellectual function rather than improvement after surgery. Seizure-free and ASM-free patients reach higher FSIQ gains. More research is needed to evaluate individual changes after specific surgery types and their effect on long-term follow-up.

**Supplementary Information:**

The online version contains supplementary material available at 10.1007/s00415-023-12002-8.

## Introduction

Epilepsy is one of the most common neurological disorders in childhood. Developmental delay, cognitive deficits, and psychosocial comorbidities represent major challenges for children with epilepsy, resulting in a substantial impairment of quality of life (QoL) besides seizure activity [1]. Cognitive impairment in children with epilepsy occurs in up to 17% of cases compared to 1.7% in the general population and may rise to 70% in children with intractable epilepsy [2, 3]. A worse cognitive status is also strongly associated with lower psychosocial outcomes in adulthood [4]. The most relevant factor for impaired cognition in epilepsy is most likely etiology as it determines age at epilepsy onset, seizure frequency, and ASM load [5, 6]. Overall, 20–30% of patients do not become seizure-free despite the use of 2–3 ASM and these patients should be evaluated whether they are candidates for epilepsy surgery [7].

Epilepsy surgery has been shown to be an effective and safe treatment for drug-resistant epilepsy with seizure-freedom rates exceeding 70% 2 years post-surgery and complication rates reported to be less than 5% [8–12]. Approximately 25% of patients with focal epilepsy qualify for surgery [13]. Surgical approaches can include the resection of the epileptogenic zone or anatomical interruption of pathways of seizure spread, although the results for the latter are less favorable [14]. Surgery can minimize seizure activity and can also stop or even reduce the progress of cognitive degradation. Improvement of IQ scores after epilepsy surgery has been shown especially in subgroups in whom ASM could be tapered after the surgical approach [3, 15]. Taking into account the multiple individual variables, this presents a challenging task [3, 16]. Comprehensive data regarding cognitive outcomes after surgical intervention are scarce, and knowledge of the effects on (cognitive) development is still little. We aimed to determine postoperative outcomes of cognitive development by systematically reviewing the existing literature.

## Methods

### Standard of reporting

This systematic review was conducted according to the “Preferred Reporting Items for Systematic Reviews and Meta-Analysis” (PRISMA) guideline.

### Search strategy

The electronic databases PubMed and Web of Science were searched using the keywords “epilepsy,” “surgery,” “children,” and “cognition.” An advanced search was conducted using “Medical subject headings” (MeSH). The search was conducted on December 3, 2021, for PubMed and on January 11, 2022, for Web of Science. Studies had to be written in English and published after the year 2000 (Online Resource 1).

### Inclusion criteria

Studies were included if the mean age of the study population was below 18 years, and the standard deviation suggested that the majority of the population was younger than 18 years old. Studies were also included if it was possible to differentiate between patients who were older or younger than 18 years within the study. In that case, only the group younger than 18 years was included in this review. All children needed to be diagnosed with medically intractable epilepsy and, except for the control groups, needed to have undergone epilepsy surgery. At least five patients needed to have a preoperative and postoperative cognitive assessment. A study was included if the assessed intervention included temporal or extratemporal resection or hemispheric procedures such as hemispherectomy or functional hemispherotomy. Studies were excluded if more than five percent of the assessed interventions were of palliative purpose such as vagus nerve stimulation, corpus callosotomy, or anterior thalamic stimulation. Studies were included if they reported FSIQ, DQ, or pooled IQ/DQ baseline and outcome after surgery on an individual or group level (mean). If study populations were overlapping in at least two studies (i.e., different studies from the same authors/centers), only the study with the largest population was included.

### Data extraction

The primary outcome variable was the full-scale intelligence quotient after epilepsy surgery. The secondary outcome variables were developmental quotient, or pooled IQ/DQ after epilepsy surgery. The following data were extracted from the studies: author, year and country of publication, size of the population, size of the control group (if available), age at epilepsy onset, age at surgery, type of intervention, the method of IQ measurement, duration of postsurgical follow-up, mean change of FSIQ, DQ, IQ/DQ, baseline FSIQ, DQ, IQ/DQ, postoperative FSIQ, DQ, IQ/DQ, percentage of seizure-free patients, postoperative FSIQ, DQ, IQ/DQ of seizure-free patients and postoperative FSIQ, DQ, IQ/DQ of not seizure-free patients. The following variables were assessed for prediction: mean age at surgery, age at epilepsy onset, preoperative IQ, duration of epilepsy to surgery, and percentage of seizure-free patients within each cohort.

### Statistical analysis

For between-group differences (preoperative FSIQ vs. postoperative FSIQ, seizure-free at follow-up vs. not seizure-free at follow-up, ASM-free at follow-up vs. not ASM-free at follow-up), meta-analysis was conducted using IBM SPSS 28 and Review Manager 5.4 software. The mean difference and confidence intervals were calculated using a random-effects model integrating heterogeneity between studies. Heterogeneity between studies was assessed using I^2^. For meta-analysis with a small sample size, Hedges’ g was included to assess the effect strength. Meta-analysis was visualized using forest plots. A leave-one-out analysis was conducted for post hoc analysis to address whether one study or a set of studies was influential on the pooled estimate. The risk of bias was assessed using funnel plots. If standard deviation was not reported in the study, it was calculated using a method that has been shown to be reliable [17, 18]. If the mean standard deviation had to be calculated from study subgroups, a method from the Cochrane Handbook was used [19]. If standard deviation could not be calculated with these methods, the study was not included in the meta-analysis. Bubble plots were used to analyze correlations. For studies reporting FSIQ outcomes and seizure outcomes on an individual level, an unpaired *t* test was conducted. A paired *t* test meta-analysis was included to compare the surgical groups to the control groups’ change of FSIQ. Paired t tests were also conducted for sensitivity analysis of the control group comparison. The Department of Medical Information Processing, Biometry, and Epidemiology (IBE) of Ludwig-Maximilian-University Munich provided advisory support for the statistical analysis.

### Study quality

The studies were assessed using the Effective Public Health Practice Project (EPHPP) quality rating tool [20]. The tool allows evaluation of a study based on the aspects of “selection bias,” “study design,” “confounders,” “blinding,” “data collection method,” and “withdrawals and dropouts.” For each category, the studies are either rated as strong, moderate, or weak. The results were then used to derive a global score.

## Results

### Search strategy

**Fig. 1:**
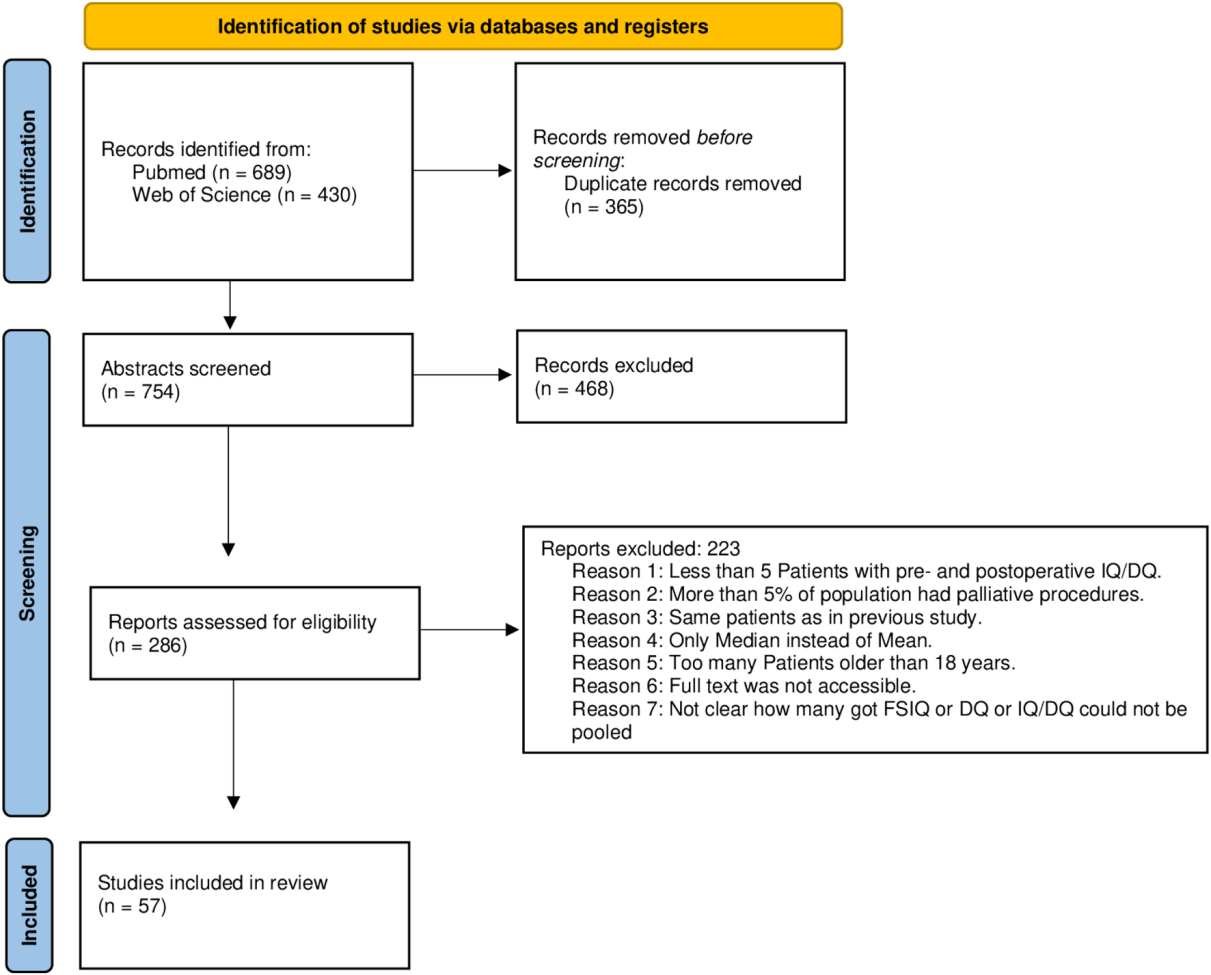
PRISMA flowchart of study selection

The outlined search strategy yielded 689 papers in PubMed and 430 in Web of Science (Fig. 1). After 365 duplicates were removed, 754 papers remained to be screened. Screening of the abstracts resulted in 286 papers which subsequently were to be assessed for eligibility. Sixty papers met the inclusion criteria. Five of these studies were by the same research groups and therefore the study populations were overlapping [21–25]. In these cases, the study version with the larger population was included, resulting in 57 remaining studies.

### Study characteristics

The studies reported on the neuropsychological outcome of 2593 children following epilepsy surgery. Sample sizes ranged from 5 to 301 patients. The mean duration of follow-up was 2.8 years (± 2.1; range 1 month–21 years). Thirty-two studies reported cognitive change at the group level and 25 at the individual level. Forty-four studies reported full-scale IQ change data, 11 reported DQ change data, and 5 reported pooled IQ/DQ change data (Online Resource 2).

### Patients’ demographics

51% (*n* = 1324) of the population was male. The overall mean age at surgery was 9.2 years (± 3.4; range 2.4 months–19.8 years). The mean age at epilepsy onset was 4.2 years (± 2.4; range 0–16.9 years). All patients had medically focal refractory epilepsy. The mean duration from epilepsy onset to surgery was 5.3 years (± 2.2; range 0–19 years).

### Cognitive outcome

**Fig. 2:**
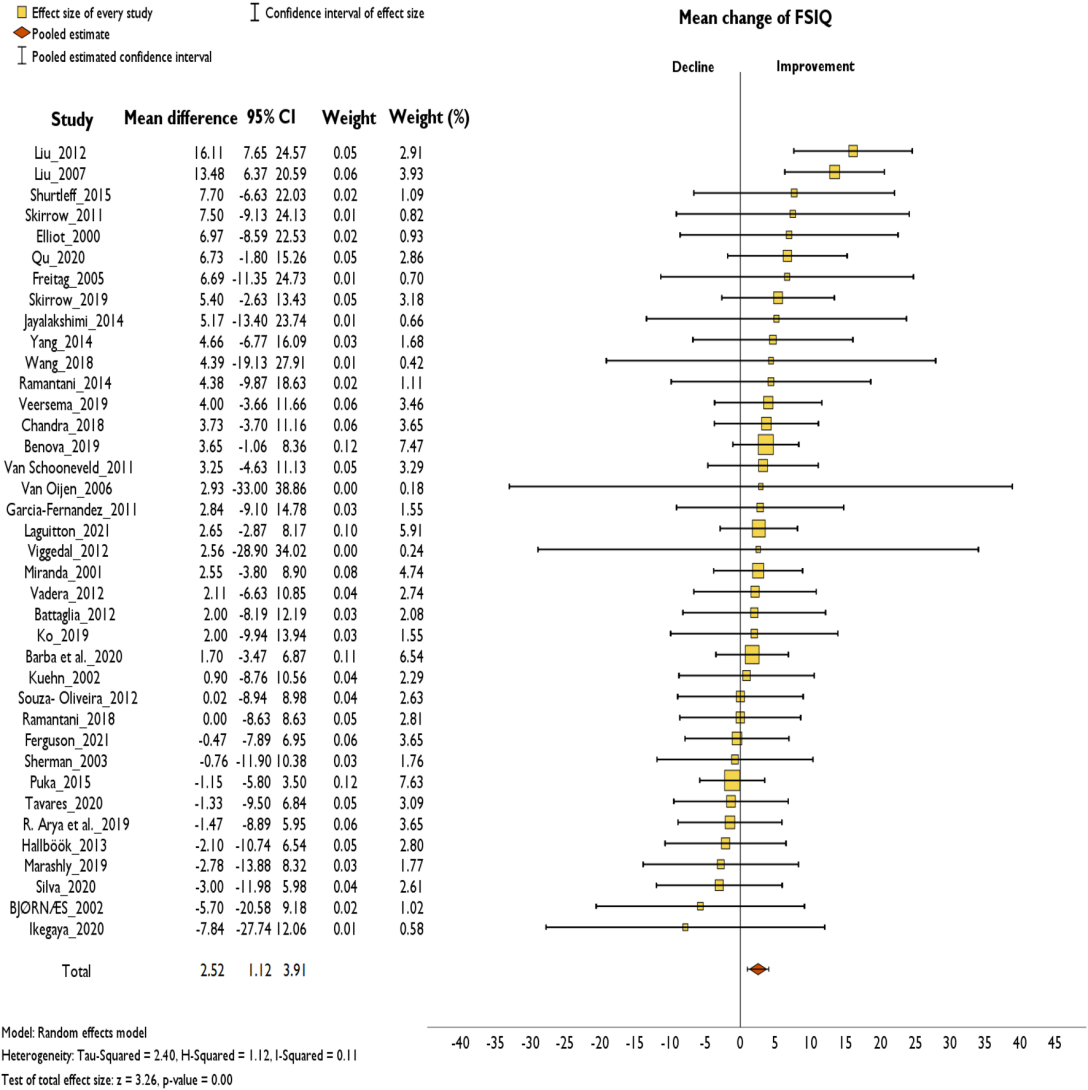
Random-effects meta-analysis of mean change of FSIQ over a mean of 2.77 years (range 0.1–21 years)

Across the studies reporting the change of FSIQ at the individual level 207 (63%) of 327 children improved in cognition after surgery, 13 remained stable and 107 deteriorated. Across the studies reporting the change of DQ at the individual level, 38 (48%) of 80 children improved in cognition after surgery, 1 remained stable and 41 deteriorated. Across the studies reporting the change of pooled IQ/DQ at the individual level 20 (56%) of 36 children improved in cognition after surgery, 3 remained stable and 13 deteriorated. On a group level, of the 44 studies reporting on FSIQ, 31 (71%) showed improvement, 1 yielded a stable outcome and 12 showed deterioration (Online Resource 2). Six studies could not be included within the forest plot analysis as they did not report on standard deviation or standard deviation that could not be calculated using the methods mentioned above. Meta-analysis was conducted using a random-effects model. It showed a significant pooled estimate of + 2.52 FSIQ points (95% CI 1.12–3.91, *p* < 0.001). I2 test yielded low heterogeneity (I2 = 0.11) (Fig. 2). Post hoc analysis showed that no single study had noticeably strong influence on the pooled estimate or heterogeneity with the overall effect size ranging from 2.08 to 2.88 (Online Resource 3). Omitting studies in which the age at surgery ranged above 18 years (*n* = 4) did not change the overall effect size drastically (mean difference = 2.6, 95% CI 1.12–4.07) [26–29]. Of the six studies that are not included in the forest plot, five showed improved FSIQ and one showed minimal decline resulting in a mean change of FSIQ of + 3.96 (± 2.35, range − 0.01 to 6.43).

Of the studies reporting on DQ, 7 (64%) showed improvement in DQ after surgery, while 4 showed worsening. Meta-analysis could only be conducted for 7 studies because for 4 studies standard deviation was not reported or could not be calculated. The pooled mean difference of DQ was + 1.47 (95% CI − 6.5 to 9.5). Of the studies reporting on pooled IQ/DQ, two showed improvements, while three showed declines. Meta-analysis could only be conducted for 3 studies for the same reasons mentioned above. The pooled mean difference of IQ/DQ was 0.73 (95% CI − 4.8 to 6.2). However, in neither of these two groups, the pooled effect size reached statistical significance.

### Seizure-free vs. not seizure-free

**Fig. 3:**
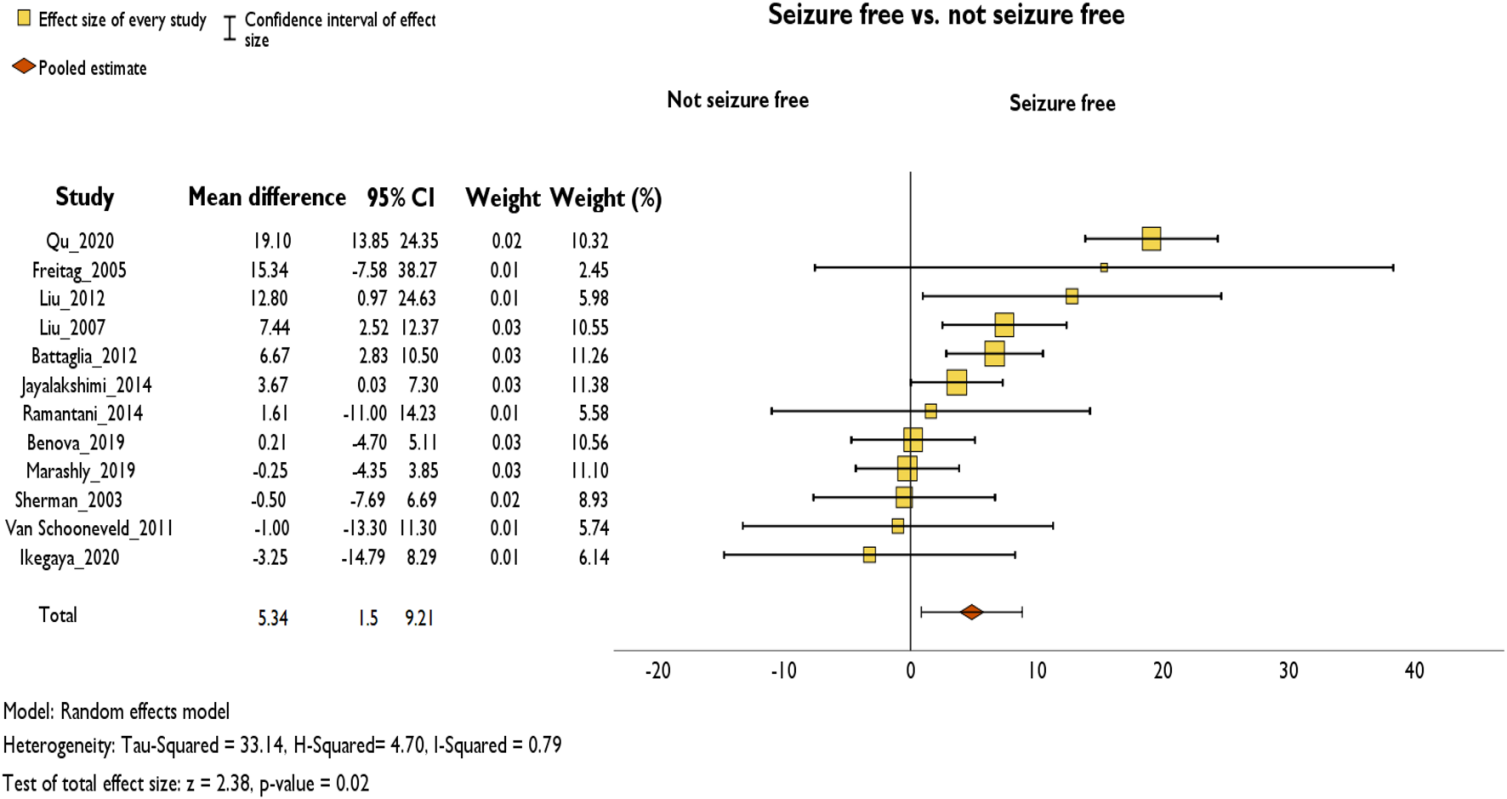
Random-effects meta-analysis of mean difference of ΔFSIQ between seizure-free and not seizure-free patients at follow-up

The overall pooled delta FSIQ was 5.34 (95% CI 1.5–9.21, *p* = 0.007, Hedges’ *g*: 0.7) points higher in the seizure-free at follow-up group (+ 5.58 ± 8.27) compared to the non-seizure-free group + 0.23 ± 5.65) (*n* = 12 studies available for this subgroup analysis) (Fig. 3). Post hoc analysis showed that one study had an increasing effect on heterogeneity in the meta-analysis. Also, the overall effect size changed noticeably when this study was omitted (mean difference = 3.9, 95% CI 1.12–6.70) (Online Resource 3) [30].

For studies that reported on an individual level, Table 1 shows the difference between patients who were seizure-free at follow-up and those who were not. An unpaired t test showed that patients who were seizure-free at follow-up had a significantly higher change of FSIQ (+ 4.92 ± 11.16; range – 30 to 39) than patients who still had seizures (+ 1.94 ± 9.28; range − 20 to 29, *p* = 0.004); *t*(42) = 2.67; *d* = 0.28 (Table 1).

**Table 1:** Individual data of seizure-free vs. not seizure-free patients at follow-up

Seizure-free at follow-up			Not seizure-free at follow-up		
FSIQ delta mean	FSIQ Pre-OP mean	FSIQ Post-OP mean	FSIQ Delta mean	FSIQ Pre-OP mean	FSIQ Post-OP mean
4.92 (± 11.16; − 30 to 39)	80.13 (± 20.9; 23–124)	84.86 (± 21.22; 19–140)	1.94 (± 9.28; − 20 to 29)	73.91 (± 18.70; 42–112)	75.93 (± 17.56; 35–112)

### ASM free vs. not ASM free

**Fig. 4:**
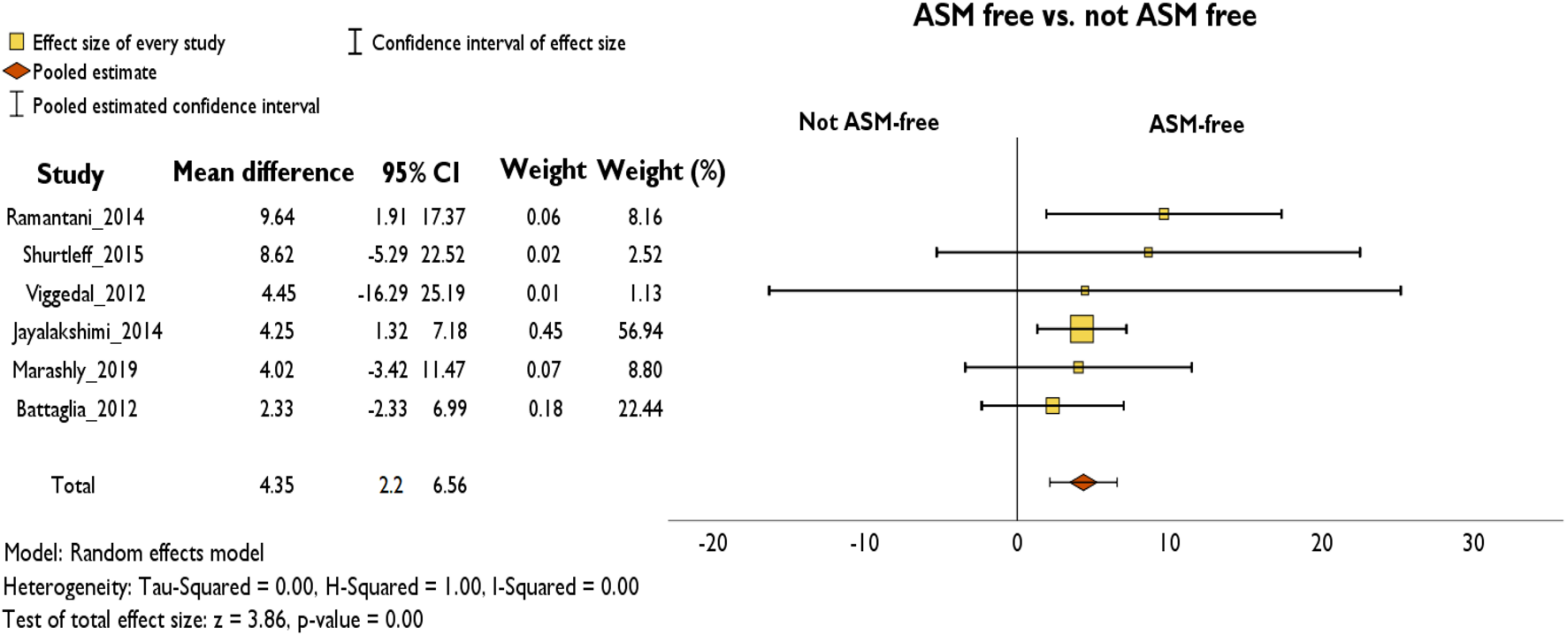
Random-effects meta-analysis of mean difference of ΔFSIQ between ASM-free and not ASM-free patients at follow-up

Comparing patients who became free of anti-seizure medication to patients who did not, the ASM-free patients showed significantly better cognitive outcomes (mean FSIQ gain in ASM-free patients: + 6.37 ± 3.80; in not ASM-free patients: + 2.01 ± 2.41). The overall pooled delta FSIQ was + 4.35 (95% CI 2.2–6.6, *p* < 0.001, Hedges’ *g*: 0.7) points higher in the ASM-free group (Fig. 4). Post hoc analysis showed that no single study had a noticeably strong influence on the pooled estimate or on heterogeneity (Online Resource 3).

### Factors associated with the change of FSIQ

**Fig. 5:**
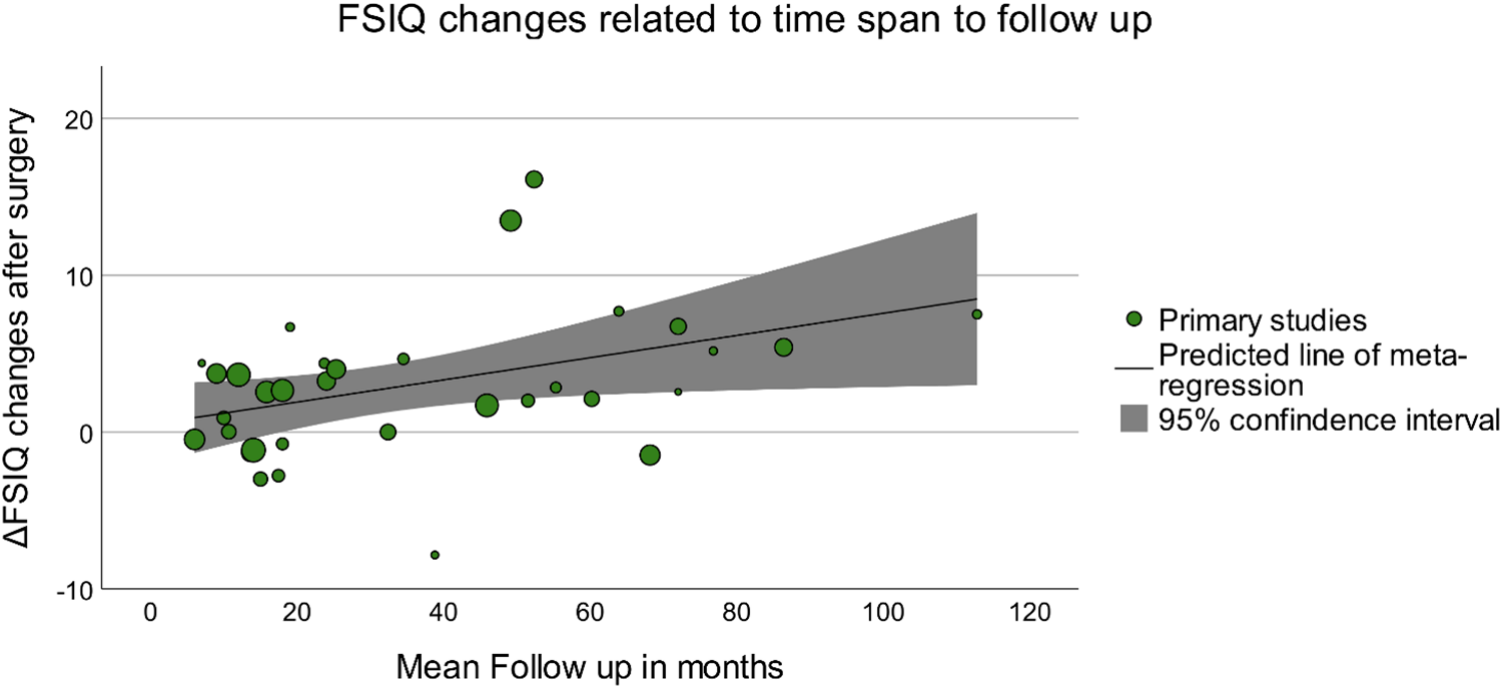
Bubble plot of FSIQ changes related to time span to follow-up

The test of heterogeneity yielded low heterogeneity (*I*^2^ = 0.11). Therefore, surgery seems to have had the biggest effect on cognitive improvement. No correlation between the change of FSIQ and the factors of age at surgery, epilepsy duration to surgery, and preoperative FSIQ could be shown. However, the bubble plot of duration of follow-up vs. delta FSIQ indeed indicated a higher chance of FSIQ in studies with longer follow-up periods (R2 = 7.1%, *p* = 0.001) (Fig. 5).

### Comparison with non-surgical controls

Four studies compared the outcome of the surgical group to a non-surgical control group. Three of them reported a change of FSIQ, while one reported pooled IQ/DQ outcome. In all four studies, the surgical group had better outcomes than the control group. A paired t test showed significantly higher change of FSIQ in the surgical group (*M* = 4.05 ± 4.30) than in the control group (*M* = − 1.17 ± 2.23); *t*(2) = 2.98, *p* = 0.048 [31–33]. However, sensitivity analysis yielded no significant increase of FSIQ in the surgical group (*p* = 0.12) nor a significant decrease in the control group (*p* = 0.23). The only study reporting on pooled IQ/DQ showed a mean gain of 1.3 points in the surgical group and a decline of − 2.6 points in the control group [34].

### Study quality

A funnel plot showed evenly distributed studies which indicates a low risk of bias (Online Resource 4). Twenty-six of 57 studies were given a strong rating, 30 studies were given a moderate rating, and one was given a weak rating. All studies were ranked as “moderate” in the category “selection bias.” In the category “study design,” three studies were clinical controlled studies and therefore given a strong rating. In the category “confounders,” 22 studies were given a strong rating. Twenty-six were given a weak rating as the studies did not report on confounders or did not adjust them. Two studies were rated strong in the category “blinding.” The remaining studies were ranked moderate. In the category “data collection,” 49 studies were ranked as strong. Four studies were given a moderate ranking because the authors added individual approaches to valid methods. The other four studies were given a weak ranking. The category “withdrawals” did not apply to most studies as most data were collected retrospectively. All studies for which it was applicable were ranked as strong.

## Discussion

### Cognitive outcome

The majority of studies revealed an increase in FSIQ, and the mean increase was 2.52 FSIQ points. FSIQ strongly reflects cognitive performance. However, it is more challenging to define which amount of FSIQ changes correlate with a reasonable clinical change of cognitive function [35]. Studies investigating IQ changes after epilepsy surgery most commonly define an increase of at least 10 IQ points as a clinically meaningful improvement of cognitive function [3, 15]. Thus, the mean gain of 2.52 FSIQ points detected in this meta-analysis rather reflects sustainability than improvement of intellectual function after surgical treatment of children and adolescents with epilepsy. Nonetheless, studies investigating neuropsychological outcomes after epilepsy surgery reported clinical improvement besides only little statistical changes [36, 37]. As many patients suffer from cognitive decline before surgery, stable FSIQ after surgery would already be equivalent to a relevant improvement in cognitive development. One study that was included in the meta-analysis found that patients experienced positive outcomes clinically, although they did not show statistically significant change [38]. The latter group also concluded that in order to reach significant postoperative improvement of FSIQ, patients who show preoperative cognitive stagnation would have to return to much faster cognitive progress than observed in typical development. As this meta-analysis found improvement, one can assume that some patients experienced less severe preoperative cognitive decline than others. Some of the studies in this review reported a decline in FSIQ. The study which reported the worst outcome included only a small number of patients who suffered from severe epilepsy with daily seizures before surgery and most of the patients with FSIQ decline had an unfavorable seizure outcome [39].

Meta-analysis of preoperative vs. postoperative DQ resulted in slight mean improvement, although statistical analysis did not reach significance. The same applies to the outcome of pooled IQ and DQ values. Only seven studies reporting on DQ outcomes and only three studies reporting on pooled IQ and DQ outcomes could be included in the meta-analysis. We found too few studies reporting on DQ. This might either be due to a lack of studies or due to our search strategy as we did not search for the term “DQ” specifically. Full-scale IQ does not represent children with severe cognitive impairment as well as DQ [40]. Therefore, more studies on DQ need to be analyzed to attain an appropriate pooled estimate.

### Seizure-free vs. not seizure-free

Seizure freedom predicted better cognitive outcomes. This is in line with findings that continuous seizure activity may have detrimental effects on cognitive networks [41]. In addition, lack of seizure freedom after epilepsy surgery may point to a more diffuse epileptogenic zone and lesion which might itself contribute to the interference with cognitive networks [42]. Lastly, seizure freedom after epilepsy surgery usually leads to ASM tapering, which has been associated with significant gains in total IQ [43]. In sensitivity analysis, one study had a big influence on the pooled estimate as well as on the reduction of I2. The study population was analyzed further to assess why the discrepancy was so high between seizure-free and not seizure-free patients in this case. The additional analysis showed that children who did not become seizure-free at follow-up showed a higher duration of epilepsy in the past than children who became seizure-free [30]. Therefore, this confounder must be object to research in the future.

### ASM free vs. not ASM free

Patients who were without ASM after epilepsy surgery showed higher rates of FSIQ improvement than patients who were still treated with ASM. Less favorable cognitive outcomes in patients still receiving ASM after surgery are most likely due to the ongoing seizures or the detrimental effects of some ASM [44]. The latter observation is in line with previous findings that IQ improves after ASM withdrawal following pediatric epilepsy surgery [43].

### Age at surgery

Due to very low I2, surgery appears to explain the effect of cognitive improvement to the biggest extent. However, many studies that were included in the review reported better cognitive outcomes for patients who underwent epilepsy surgery at a younger age [45–49]. This raises the question of why the bubble plot did not suggest this relation between cognitive outcome and the age at surgery in this review. The effect might be stronger among very young children since surgery would stop developmental stagnation at a more pivotal stage of brain development. The missing relation of FSIQ changes and age at surgery might be unmasked if different age groups are looked at more precisely. The extracted data did not allow that kind of group analysis in this review as the distraction to certain different age groups would have yielded low numbers of patients in each distinct group and thus weak power to reveal any relationships.

### Duration of follow-up

The bubble plot indicated that studies with a longer follow-up period showed a higher rate of improvement of FSIQ. However, R2 appeared to be low. This might occur due to the very small number of long-term studies. Most studies only followed the patient for a short-term period. The few studies that followed up with the patients in a long-term perspective showed a higher change of FSIQ. A tendency toward greater cognitive improvement after long follow-up is apparent. This correlation is mirrored by individual findings of studies included in our review [31, 50]. Some longitudinal studies in this review reported on multiple follow-up assessments. By determining a mean value of the duration of follow-up for each of these studies, within-study effects of longer follow-up may have been neglected, and thereby data might have been skewed. More long-term studies are needed to observe this relationship.

### Comparison with non-surgical controls

Four of the studies we included in the review compared a surgical group to a group treated only with ASM. Three of these studies reported higher rates of IQ improvement in the surgical group. Due to the small number of these studies, statistical analysis does not have a lot of power. However, one randomized controlled study, which was not included in the review (> 5% of surgical procedures concerned corpus callosotomies), showed no difference in IQ improvement between the surgical and non-surgical groups despite higher rates of seizure freedom and improved quality of life and behavior in the surgical group [9]. One of the studies evaluating FSIQ outcomes that we included in our meta-analysis found no difference between the surgical group and the control group [33]. However, this study considered only outcomes 18 months after surgery. This reflects our findings of a correlation between cognitive outcome and duration of follow-up. Two of the four studies included in the review evaluated the patients from a long-term perspective. One evaluated the patients at a mean of seven years after surgery and one at a mean of nine years after surgery [31, 32]. Both found improvement in FSIQ in the surgical group, while this improvement was not apparent in the control group. Another controlled longitudinal study reported on a pooled IQ/DQ value. It found better cognitive outcomes in the surgical group compared to the control group after a follow-up period of 24 months. The study also assessed the patients at 12 months after surgery. At that point, cognitive improvement was not yet seen [34]. This also underlines the relation between cognitive outcome and duration of follow-up.

### Limitations

Not all studies which met inclusion criteria could be included in the meta-analysis. This was mainly due to a lack of reporting of statistic parameters such as standard deviation or standard error. In some cases, missing variables could be estimated using the methods mentioned above. If this was not the case, studies were excluded from the meta-analysis. Therefore, the pooled estimate does not represent all included studies. Of 44 studies reporting on FSIQ outcomes, six studies could not be incorporated in the comparison between pre- and postoperative FSIQ [22, 29, 50–53]. Since these six studies added up to a mean change of FSIQ of 3.96, it can be assumed that meta-analysis would have yielded a higher overall pooled estimate if the studies were included. Homogenous reporting of statistic parameters will be crucial in future studies.

By comparing one preoperative to one postoperative value, this paper neglected the dynamics of FSIQ development. Some studies reported a negative mean difference in FSIQ after surgery. However, some of them additionally described a decline in FSIQ before surgery. In many cases, downward trend could be reversed or at least stagnation could be stopped through epilepsy surgery [32]. In some studies, this was only the case in patients who became seizure-free after surgery [39, 54]. This indicates that a pooled mean difference of pre- and postoperative FSIQ might not be differentiated enough to represent the effectiveness of epilepsy surgery appropriately. Longitudinal studies are compelling to analyze this effect.

In this meta-analysis, we decided to search for IQ as the primary outcome variable to attain an objective pooled estimate. Consequently, the review did not differentiate between several subcategories of cognition. We found various studies indicating that surgery might affect some subcategories more than others. However, the studies showed heterogeneous outcomes. For example, children with Rasmussen encephalitis showed improvement in verbal comprehension five years after hemispherotomy [55]. On the other hand, visual memory seems to decline in many children after surgery [56]. However, another study found that visual memory improves in the long-term perspective. The same study also found that early surgery is associated with higher chances of improvement of cognitive domains [57]. Language and memory improved after three years in one study [58]. Observing cognitive subcategories in a more differentiated manner will be essential, so individual risks and chances can be assessed. Hence, homogenous reporting of cognitive domains will be crucial. The meta-analysis of seizure-free vs. not seizure-free patients at follow-up yielded high heterogeneity. Therefore, further stratification of the analysis is necessary. However, that is not feasible in this case due to the small sample size. Also, since seizure freedom is associated with freedom of ASM, higher FSIQ in seizure-free and ASM-free patients may be aiming at the same subject. The included studies did not provide sufficient data on the location of the lesion. Therefore, we could not evaluate in how far this influenced the cognitive outcome. Furthermore, we assume that the cognitive outcome is associated with the socio-professional status of the parents. This has not been evaluated by the included studies. Consequently, this points to a question that should be addressed in future research.

## Conclusions

The present review indicates that there is a mean gain in FSIQ and DQ in children with medically intractable epilepsy after surgery. Seizure-free and ASM-free patients reach higher FSIQ gains. More research is needed to evaluate individual changes after specific surgery types, their effect on long-term follow-up, and the association of gaining IQ scores to clinically meaningful cognitive improvement.

### Supplementary Information

Below is the link to the electronic supplementary material.

Supplementary file1 (DOCX 22 KB)

Supplementary file2 (DOCX 119 KB)

Supplementary file3 (DOCX 20 KB)

Online Resource 4: Funnel plot reveals evenly distributed studies, indicating a low risk of bias (TIFF 16063 KB)

## Data Availability

Most of the analyzed data were included in the manuscript or supplemental material. Data used in this study are also available upon reasonable request from the corresponding author.

## Abbreviations

FSIQ: Full-scale intelligence quotient
DQ: Developmental quotient
ASM: Anti-seizure medication
CI: Confidence interval
SD: Standard deviation
TLE: Temporal lobe epilepsy
