# Supplementary material for: IQ changes after pediatric epilepsy surgery: a systematic review and meta-analysis
Source: J Neurol. 2023 Sep 28;271(1):177–87. doi: 10.1007/s00415-023-12002-8 (PMC10770207; doi:10.1007/s00415-023-12002-8)
Supplement: Supplementary file 1 — Supplementary file1 (DOCX 22 KB) [file 415_2023_12002_MOESM1_ESM.docx]

|  | Table e-1: Search strategy |  |
| --- | --- | --- |
|  | Pubmed 03.12.2021 |  |
| # | **Combination** | **Results** |
|  | **Population: Epilepsy** |  |
| 1. | (epilep*[tiab] OR seizure*[tiab] OR “drug resistant epilep*”[tiab] OR “medication resistant epilep*”[tiab] OR “intractable epileps*”[tiab] OR “refractory epileps*”[tiab]) | 213.121 |
| 2. | (“Epilepsy/surgery”[Majr] OR "Seizures/surgery"[Majr] OR "Drug Resistant Epilepsy/surgery"[Majr] OR "Drug Resistant Epilepsy"[Mesh] OR "Seizures/prevention and control"[Majr] OR "Drug Resistant Epilepsy/psychology"[Majr]) | 11.306 |
| 3. | 1 OR 2 | 214.071 |
|  | **Population: Children** |  |
| 4. | (child*[tiab] OR pediatr*[tiab] OR infant*[tiab] OR adolescent*[tiab] OR teen*[tiab]) | 2.151.386 |
| 5. | ("Adolescent"[Mesh] OR "Child"[Mesh] OR "Child, Preschool"[Mesh] OR "Infant"[Mesh]) | 3.774.997 |
| 6. | 4 OR 5 | 4.303.029 |
|  | **Intervention: Surgery** |  |
| 7. | (surg*[ti] OR operat*[ti] OR resect*[ti] OR transect*[ti] OR decortication[ti] OR lobectom*[ti] OR hemispherectom*[ti] OR hemispherotom*[ti] OR lesionectomy[ti] OR “epilepsy surg*”[ti]) | 927.393 |
| 8. | (Hemispherectomy[Mesh] OR “Anterior temporal lobectomy”[Mesh] OR "Neurosurgical Procedures/methods"[Mesh]) | 55.923 |
| 9. | 7 OR 8 | 969.895 |
|  | **Outcome: Cognition** |  |
| 10. | (Cognition[tiab] OR intellectual[tiab] OR cognitive[tiab] OR “intelligence quotient”[tiab] OR IQ[tiab] OR neuropsychological[tiab] OR mental[tiab] OR intelligence[tiab]) | 899.904 |
| 11. | (cognition[Majr] OR "Social Cognition"[Mesh] OR "Cognitive Dysfunction/surgery"[Mesh] OR "Intelligence"[Majr] OR "Cognition/physiology"[Mesh]) | 167.895 |
| 12. | 10 OR 11 | 981.520 |
|  | **Combined Sets** |  |
| 13. | 3 AND 6 AND 9 AND 12 | 908 |
|  | **Limits** |  |
| 14. | Publication date from 2000/01/01 |  |
| 15. | English |  |
| 16. | 13 AND 14 AND 15 | 689 |
|  | Web Of Science 11.01.2022 |  |
| # | **Combination** | **Results** |
|  | **Population: Epilepsy** |  |
| 1. | TI=(epilep* OR seizure* OR “drug resistant epilep*” OR “medication resistant epilep*” OR “intractable epileps*” OR “refractory epileps*”) | 158.188 |
| 2. | AB=(epilep* OR seizure* OR “drug resistant epilep*” OR “medication resistant epilep*” OR “intractable epileps*” OR “refractory epileps*”) | 158.714 |
| 3. | 1 OR 2 | 240.703 |
|  | **Population: Children** |  |
| 4. | **TI=(child* OR pediatr* OR infant* OR adolescent* OR teen*)** | 1.580.244 |
| 5. | AB=(child* OR pediatr* OR infant* OR adolescent* OR teen*) | 1.665.706 |
| 6. | 4 OR 5 | 2.418.631 |
|  | **Intervention: Surgery** |  |
| 7. | TI=(surg* OR operat* OR resect* OR transect* OR decortication OR lobectom* OR hemispherectom* OR hemispherotom* OR lesionectomy OR “epilepsy surg*”) | 1.243.710 |
|  | **Outcome: Cognition** |  |
| 8. | TI=(Cognition OR intellectual OR cognitive OR “intelligence quotient” OR IQ OR neuropsychological OR mental OR intelligence) | 560.349 |
| 9. | AB=(Cognition OR intellectual OR cognitive OR “intelligence quotient” OR IQ OR neuropsychological OR mental OR intelligence) | 1.182.393 |
| 10. | 8 OR 9 | 1.422.753 |
|  | **Combined Sets** |  |
| 11. | 3 AND 6 AND 7 AND 10 | 501 |
|  | **Limits** |  |
| 12. | Publication date from 2000/01/01 |  |
| 13. | English |  |
| 14. | 13 AND 14 AND 15 | 430 |
| 15. | 16. PubMed AND 14. WoS | 1.119 |
| 16. | 365 Duplikate entfernt | 754 |
| 17. | Davon PubMed | 689 |
| 18. | Davon WoS | 65 |
