# Supplementary material for: IQ changes after pediatric epilepsy surgery: a systematic review and meta-analysis
Source: J Neurol. 2023 Sep 28;271(1):177–87. doi: 10.1007/s00415-023-12002-8 (PMC10770207; doi:10.1007/s00415-023-12002-8)
Supplement: Supplementary file 2 — Supplementary file2 (DOCX 119 KB) [file 415_2023_12002_MOESM2_ESM.docx]

## Table e-2: Study characteristics

| Study | N | Male | Female | Age at onset in months [Mean (Range)] | Age at surgery in months [Mean (Range)] | Duration of Epilepsy in months [Mean (Range)] | ΔFSIQ; ΔDQ; ΔIQ/DQ |
| --- | --- | --- | --- | --- | --- | --- | --- |
| Bajer et al. 2020^1^ | 75.00 | 46 | 29 | 23.88 | 83.88 (10.44-237.66) | 60.00 | ΔFSIQ: 4.44 ΔDQ:  18.00 |
| Barba et al. 2021^2^ | 114.00 | 63 | 51 | 61.20  (SD: 51.6) | 109.20 (SD: 54) | 48.00  (SD: 40.8) | ΔFSIQ: 1.70 |
| Battaglia 2012^3^ | 12.00 | 8 | 4 | 104.50 (60-156) | 120.83 (72-197) | 16.42 (3-53) | ΔFSIQ:  2.00 |
| Benova 2019^4^ | 135.00 | 72 | 63 | 55.93 (0-193.2) | 114.26 (3.6-214.8) | 58.33  (1.2-189.6) | ΔFSIQ: 3.65 ΔDQ: -4.00 |
| BJØRNÆS 2002^5^ | 13.00 | 8 | 5 |  | 168.00 (SD: 31.2) |  | ΔFSIQ: -5.70 |
| Boshuisen 2015^6^ | 301.00 | 147 | 154 |  | 115.20 (SD: 54) |  | ΔIQ/DQ:  -1.60 |
| Chandra 2018^7^ | 59.00 | 42 | 17 | 33.3 (0.03–168) | 80.34 | 47.04 (SD: 14.88) | ΔFSIQ: 3.73 |
| Datta  2011^8^ | 57.00 | 29 | 28 | 135.52 (33-203) | 140.00 (36-204) | 5.91  (1-15) | ΔFSIQ:  -0.01 |
| De Knegt 2020^9^ | 79.00 | 45 | 34 | Median:  46.80 (IQR: 18-87.6) | Median: 145.20 (114-168) | Median:  73.30 (44.4-118.8) | ΔFSIQ: 3.30 |
| Elliot 2000^10^ | 13.00 | 6 | 7 | 90.00 (9-174) | 164.64 (129-210) | 74.64 | ΔFSIQ:  6.97 |
| Ferguson 2021^11^ | 41.00 | 25 | 16 | 62.40 (SD: 43.2) | 126.00 (SD: 32.4) | 60.00 (SD: 42) | ΔFSIQ:  -0.47 |
| Freitag 2005^12^ | 12.00 | 5 | 7 | 20.67 (3-52) | 59.75 (38-81) | 39.08 (6-67) | ΔFSIQ:  5.38 ΔDQ: 15.00 |
| Garcia-Fernandez 2011^13^ | 19.00 | 13 | 6 | 80.33 (2.4-180) | 127.77 (24-204) | 47.44  (6-132) | ΔFSIQ:  2.84 |
| Hallböök 2013^14^ | 45.00 | 26 | 21 | Median:  24.00 (0-162) | Median:  96.00 (6-224.4) | Median:  50.40 (6-218.4) | ΔFSIQ:  -2.11 |
| Ikegaya  2020^15^ | 15.00 | 5 | 10 | 14.20 (1-77) | 74.73 (4-163) | 60.53  (3-162) | ΔFSIQ:  -7.83 ΔDQ: -2.56 |
| Jakobsen 2020^16^ | 43.00 | 23 | 20 | 52.80 (SD: 34.8) | 133.20 (SD: 39.6) |  | ΔFSIQ:  4.70 |
| Jayalakshimi 2014^17^ | 6.00 | 5 | 1 | 7.83 (2-24) | 110.00 (36-192) | 102.17  (32-190) | ΔFSIQ:  5.17 |
| Kimura 2014^18^ | 17.00 | 9 | 8 | 11.42 (0.5-36) | 75.12 (18-145) | 63.69  (13-130) | ΔIQ/DQ: -1.47 |
| Ko 2019^19^ | 58.00 | 29 | 29 | 72.00 (37.2-118.8) | 122.40 | Median:  15.60 | ΔFSIQ:  2.00 |
| Kuehn 2002^20^ | 26.00 | 16 | 10 | 73.20 | 142.80 (SD: 44.4) | 69.60 (SD: 69.6) | ΔFSIQ:  0.90 |
| Laguitton  2021^21^ | 81.00 | 42 | 39 | 72.00 (1.2-192) | 168.00 (78-228) | 84.00  (12-228) | ΔFSIQ:  2.65 |
| Liang 2012^22^ | 206.00 | 112 | 94 | 43.20 | 135.60 (72-168) | 92.4  (24-168) | ΔFSIQ:  6.43 |
| Liu 2007^23^ | 21.00 |  |  |  | 121.71 (60-180) | 57.94 (22.8-132) | ΔFSIQ:  13.48 |
| Liu 2012^24^ | 9.00 |  |  | 48.13 (24-108) | 112.00 (60-156) | 63.87  (34-120) | ΔFSIQ:  16.11 |
| Loddenkemper 2007^25^ | 24.00 | 18 | 6 |  | 14.75 (3-34) |  | ΔDQ: 15.92 |
| Loddenkemper 2009^26^ | 5.00 | 1 | 4 | 53.40 (18-120) | 107.40 (59-179) | 54.00 (32-81) | ΔDQ: 9.80 |
| Maehara 2002^27^ | 14.00 | 9 | 5 | 1.95 (0-20) | 28.50 (4-77) | 26.55  (4-74.5) | ΔDQ: 7.70 |
| Marashly 2019^28^ | 14.00 | 7 | 7 | 39.62 (0-102) | 94.97  (42-144) | 58.18  (0-144) | ΔFSIQ:  -2.79 |
| Meekes 2015^29^ | 118.00 | 58 | 60 |  | Median:  116.76 | Median:  38.88 | ΔIQ/DQ: 5.30 |
| Miranda 2001^30^ | 50.00 | 21 | 29 | 72.90 (6-174) | 160.38 (77.16-219) | 86.94 (9.96-202.08) | ΔFSIQ:  2.55 |
| Ohashi  2013^31^ | 5.00 | 2 | 3 | 3.2 (0-10) | 21.80 (3-37) | 18.60  (3-34) | ΔDQ: 3.00 |
| Puka  2015^32^ | 136.00 | 62 | 74 | 80.50 | 157.30 | 81.56 | ΔFSIQ:  -1.15 |
| Pulsifier  2004^33^ | 53.00 |  |  | 39.20 | 92.80 | 53.60 | ΔIQ/DQ: -2.10 |
| Qu 2020^34^ | 15.00 | 7 | 8 | 52.64 (2.4-156) | 143.12 (78-204) | 89.33 (4.8-175.2) | ΔFSIQ:  6.37 |
| . Arya et al.  2019^35^ | 37.00 | 16 | 21 | 8.81 (SD: 13.95) | 73.92 (SD: 71.52) | 66.24 (SD: 70.44) | ΔFSIQ:  -1.47 |
| Ramantani 2014^36^ | 29.00 | 19 | 10 | 94.80 (0-184.8) | 140.40 (31.2-207.6) | 45.60  (3.6-183.6) | ΔFSIQ: 4.38 |
| Ramantani 2018^37^ | 75.00 | 39 | 36 | 51.60 (0-180) | 120.00 (3.6-216) | 68.40  (3.6-205.2) | ΔFSIQ: 0.00 |
| Roulet-Perez 2010^38^ | 9.00 | 3 | 6 | 8.09 (0.23-32) | 31.17 (4-63) | 22.89  (3.5-50) | ΔDQ: 3.33 |
| Sherman  2003^39^ | 22.00 | 11 | 11 | 56.40 (6-176.4) | 135.60 (72-187.2) | 79.20 (7.2-159.6) | ΔFSIQ:  -0.76 |
| Shurtleff  2015^40^ | 10.00 |  |  | 45.90 | 55.10 (23-77) | 9.20 (2-24) | ΔFSIQ:  7.70 |
| Sibilia 2017^41^ | 31.00 | 20 | 11 | 52.97 (2-156) | 104.77 (17-189) | 51.81 (3-165) | ΔIQ/DQ: 1.30 |
| Silva 2020^42^ | 8.00 |  |  | 40.20 (3.6-96) | 139.50 (84-180) | 99.30  (36-152.4) | ΔFSIQ:  -3.00 |
| Skirrow  2011^43^ | 42.00 | 21 | 21 | 48.12 (SD: 48) | 159.60 (SD: 37.20) | 111.48 | ΔFSIQ:  7.50 |
| Skirrow 2019^44^ | 39.00 | 21 | 18 | 75.60 (SD: 51.6) | 168.00 (72-216) | 92.40 | ΔFSIQ:  5.40 |
| Souza- Oliveira  2012^45^ | 59.00 | 41 | 18 | 53.37 (1-186) | 132.29 | 77.20  (4-189) | ΔFSIQ:  0.02 |
| Sugano 2014^46^ | 10.00 | 6 | 4 |  | 28.00 (8-98) |  | ΔDQ: 17.70 |
| Tavares 2020^47^ | 8.00 | 6 | 2 | 55.20 (10.8-96) | 129.20 (68.4-190.8) | 74.00 (19.2-102) | ΔFSIQ:  -1.33 |
| Vadera 2012^48^ | 45.00 | 25 | 20 | 45.60 (0-180) | 138.00 (18-216) | 86.00  (7-192) | ΔFSIQ:  2.11 |
| Van Oijen 2006^49^ | 69.00 | 33 | 36 | 45.60 | 110.40 (6-204) | 66.00 | ΔFSIQ:  2.93 |
| Van Schooneveld 2011^50^ | 12.00 | 3 | 9 | 37.22 (0-132) | 109.58 (45.12-171.6) | 72.36 (13.44-140.88) | ΔFSIQ:  3.25 |
| Veersema  2019^51^ | 42.00 | 24 | 18 | Median: 27.60 (0-144) | Median:  120.00 (2.4-222) | Median:  54.00  (0-204) | ΔFSIQ:  4.00 |
| Viggedal 2012^52^ | 9.00 | 5 | 4 |  | 118.53 (55.2-200.4) |  | ΔFSIQ:  2.56 |
| Villarejo-Orteg 2013^53^ | 17.00 | 12 | 5 | 20.06 (0-104) | 75.53 (8-210) | 55.47  (7-210) | ΔDQ: -2.06 |
| Wang 2018^54^ | 7 | 2 | 5 |  | 133.20  (SD: 41.76) | 50.88  (3.6-108) | ΔFSIQ:  4.38 |
| Westerveld 2000^55^ | 82.00 | 48 | 34 | 64.50 | 172.50 | 108.00 | ΔFSIQ:  1.40 |
| Yang 2014^56^ | 12.00 | 6 | 6 | 48.00 (0-132) | 147.60 (48-216) | 99.60 | ΔFSIQ:  4.67 |
| Zaroff  2005^57^ | 6.00 | 2 | 4 |  | 52.67 (29-120) |  | ΔDQ: -9.40 |
|  |  |  |  |  |  |  |  |

## References to all included studies

1. Bajer C, Hofer W, Pieper T, Kudernatsch M, Holthausen H, Staudt M. Correlates of intellectual development before and after hemispherotomy: an analysis of 75 children and adolescents. *Epileptic Disord*. Oct 1 2020;22(5):571-581. doi:10.1684/epd.2020.1193

2. Barba C, Cossu M, Guerrini R, et al. Temporal lobe epilepsy surgery in children and adults: A multicenter study. *Epilepsia*. Jan 2021;62(1):128-142. doi:10.1111/epi.16772

3. Battaglia D, Chieffo D, Tamburrini G, et al. Posterior resection for childhood lesional epilepsy: neuropsychological evolution. *Epilepsy Behav*. Feb 2012;23(2):131-7. doi:10.1016/j.yebeh.2011.11.014

4. Benova B, Belohlavkova A, Jezdik P, et al. Cognitive performance in distinct groups of children undergoing epilepsy surgery-a single-centre experience. *PeerJ*. 2019;7:e7790. doi:10.7717/peerj.7790

5. Bjørnaes H, Stabell KE, Henriksen O, Røste G, Diep LM. Surgical versus medical treatment for severe epilepsy: consequences for intellectual functioning in children and adults. A follow-up study. *Seizure*. Dec 2002;11(8):473-82. doi:10.1016/s1059-1311(02)00134-6

6. Boshuisen K, Lamberink HJ, van Schooneveld MM, et al. Cognitive consequences of early versus late antiepileptic drug withdrawal after pediatric epilepsy surgery, the TimeToStop (TTS) trial: study protocol for a randomized controlled trial. *Trials*. Oct 26 2015;16:482. doi:10.1186/s13063-015-0989-2

7. Chandra PS, Subianto H, Bajaj J, et al. Endoscope-assisted (with robotic guidance and using a hybrid technique) interhemispheric transcallosal hemispherotomy: a comparative study with open hemispherotomy to evaluate efficacy, complications, and outcome. *J Neurosurg Pediatr*. Nov 9 2018;23(2):187-197. doi:10.3171/2018.8.Peds18131

8. Datta AN, Snyder TJ, Wheatley MB, et al. Intelligence quotient is not affected by epilepsy surgery in childhood. *Pediatr Neurol*. Feb 2011;44(2):117-21. doi:10.1016/j.pediatrneurol.2010.10.011

9. de Knegt VE, Hoei-Hansen CE, Knudsen M, et al. Increase in cognitive function is seen in many single-operated pediatric patients after epilepsy surgery. *Seizure*. Oct 2020;81:254-262. doi:10.1016/j.seizure.2020.08.002

10. Elliott IM, Lach L, Smith ML. Adolescent and Maternal Perspectives of Quality of Life and Neuropsychological Status Following Epilepsy Surgery. *Epilepsy Behav*. Dec 2000;1(6):406-417. doi:10.1006/ebeh.2000.0129

11. Ferguson L, Miller M, Whiting A, et al. Cognitive outcomes following frontal lobe resection for treatment of epilepsy in children and adolescents. *Epilepsy Behav*. Sep 9 2021;124:108265. doi:10.1016/j.yebeh.2021.108265

12. Freitag H, Tuxhorn I. Cognitive function in preschool children after epilepsy surgery: rationale for early intervention. *Epilepsia*. Apr 2005;46(4):561-7. doi:10.1111/j.0013-9580.2005.03504.x

13. García-Fernández M, Fournier-Del Castillo C, Ugalde-Canitrot A, et al. Epilepsy surgery in children with developmental tumours. *Seizure*. Oct 2011;20(8):616-27. doi:10.1016/j.seizure.2011.06.003

14. Hallböök T, Tideman P, Rosén I, Lundgren J, Tideman E. Epilepsy surgery in children with drug-resistant epilepsy, a long-term follow-up. *Acta Neurol Scand*. Dec 2013;128(6):414-21. doi:10.1111/ane.12154

15. Ikegaya N, Iwasaki M, Kaneko Y, et al. Cognitive and developmental outcomes after pediatric insular epilepsy surgery for focal cortical dysplasia. *J Neurosurg Pediatr*. Aug 7 2020:1-9. doi:10.3171/2020.5.Peds2058

16. Jakobsen AV, Müller E, Uldall PV. A methodological perspective on the cognitive outcome of epilepsy surgery in children and adolescents. *Epilepsy Behav*. Oct 2020;111:107330. doi:10.1016/j.yebeh.2020.107330

17. Jayalakshmi S, Panigrahi M, Nanda SK, Vadapalli R. Surgery for childhood epilepsy. *Ann Indian Acad Neurol*. Mar 2014;17(Suppl 1):S69-79. doi:10.4103/0972-2327.128665

18. Kimura N, Takahashi Y, Shigematsu H, et al. Developmental outcome after surgery in focal cortical dysplasia patients with early-onset epilepsy. *Epilepsy Res*. Dec 2014;108(10):1845-52. doi:10.1016/j.eplepsyres.2014.09.010

19. Ko A, Kim SH, Kim SH, et al. Epilepsy Surgery for Children With Low-Grade Epilepsy-Associated Tumors: Factors Associated With Seizure Recurrence and Cognitive Function. *Pediatr Neurol*. Feb 2019;91:50-56. doi:10.1016/j.pediatrneurol.2018.10.008

20. Kuehn SM, Keene DL, Richards PM, Ventureyra EC. Are there changes in intelligence and memory functioning following surgery for the treatment of refractory epilepsy in childhood? *Childs Nerv Syst*. Jul 2002;18(6-7):306-10. doi:10.1007/s00381-002-0599-7

21. Laguitton V, Desnous B, Lépine A, et al. Intellectual outcome from 1 to 5 years after epilepsy surgery in 81 children and adolescents: A longitudinal study. *Seizure*. Oct 2021;91:384-392. doi:10.1016/j.seizure.2021.07.010

22. Liang S, Wang S, Zhang J, et al. Long-term outcomes of epilepsy surgery in school-aged children with partial epilepsy. *Pediatr Neurol*. Oct 2012;47(4):284-90. doi:10.1016/j.pediatrneurol.2012.06.014

23. Liu S, An N, Yang H, et al. Pediatric intractable epilepsy syndromes: reason for early surgical intervention. *Brain Dev*. Mar 2007;29(2):69-78. doi:10.1016/j.braindev.2006.06.009

24. Liu SY, An N, Fang X, et al. Surgical treatment of patients with Lennox-Gastaut syndrome phenotype. *ScientificWorldJournal*. 2012;2012:614263. doi:10.1100/2012/614263

25. Loddenkemper T, Holland KD, Stanford LD, Kotagal P, Bingaman W, Wyllie E. Developmental outcome after epilepsy surgery in infancy. *Pediatrics*. May 2007;119(5):930-5. doi:10.1542/peds.2006-2530

26. Loddenkemper T, Cosmo G, Kotagal P, et al. Epilepsy surgery in children with electrical status epilepticus in sleep. *Neurosurgery*. Feb 2009;64(2):328-37; discussion 337. doi:10.1227/01.Neu.0000336767.14252.76

27. Maehara T, Shimizu H, Kawai K, et al. Postoperative development of children after hemispherotomy. *Brain Dev*. Apr 2002;24(3):155-60. doi:10.1016/s0387-7604(02)00010-4

28. Marashly A, Koop J, Loman M, Lee YW, Lew SM. Examining the Utility of Resective Epilepsy Surgery in Children With Electrical Status Epilepticus in Sleep: Long Term Clinical and Electrophysiological Outcomes. *Front Neurol*. 2019;10:1397. doi:10.3389/fneur.2019.01397

29. Meekes J, van Schooneveld MM, Braams OB, et al. Parental education predicts change in intelligence quotient after childhood epilepsy surgery. *Epilepsia*. Apr 2015;56(4):599-607. doi:10.1111/epi.12938

30. Miranda C, Smith ML. Predictors of Intelligence after Temporal Lobectomy in Children with Epilepsy. *Epilepsy Behav*. Feb 2001;2(1):13-19. doi:10.1006/ebeh.2000.0142

31. Ohashi T, Kobayashi I, Ooe H, Nakagawa E. Visual cognitive function in infants with intractable epilepsy before and after surgery. *Childs Nerv Syst*. Feb 2013;29(2):255-61. doi:10.1007/s00381-012-1944-0

32. Puka K, Khattab M, Kerr EN, Smith ML. Academic achievement one year after resective epilepsy surgery in children. *Epilepsy Behav*. Jun 2015;47:1-5. doi:10.1016/j.yebeh.2015.04.062

33. Pulsifer MB, Brandt J, Salorio CF, Vining EP, Carson BS, Freeman JM. The cognitive outcome of hemispherectomy in 71 children. *Epilepsia*. Mar 2004;45(3):243-54. doi:10.1111/j.0013-9580.2004.15303.x

34. Qu XP, Qu Y, Wang C, Liu B. Long-Term Cognitive Improvement After Functional Hemispherectomy. *World Neurosurg*. Mar 2020;135:e520-e526. doi:10.1016/j.wneu.2019.12.058

35. Arya R, Roth C, Leach JL, et al. Neuropsychological outcomes after resection of cortical sites with visual naming associated electrocorticographic high-gamma modulation. *Epilepsy Res*. Mar 2019;151:17-23. doi:10.1016/j.eplepsyres.2019.01.011

36. Ramantani G, Kadish NE, Anastasopoulos C, et al. Epilepsy surgery for glioneuronal tumors in childhood: avoid loss of time. *Neurosurgery*. Jun 2014;74(6):648-57; discussion 657. doi:10.1227/neu.0000000000000327

37. Ramantani G, Kadish NE, Mayer H, et al. Frontal Lobe Epilepsy Surgery in Childhood and Adolescence: Predictors of Long-Term Seizure Freedom, Overall Cognitive and Adaptive Functioning. *Neurosurgery*. Jul 1 2018;83(1):93-103. doi:10.1093/neuros/nyx340

38. Roulet-Perez E, Davidoff V, Mayor-Dubois C, et al. Impact of severe epilepsy on development: recovery potential after successful early epilepsy surgery. *Epilepsia*. Jul 2010;51(7):1266-76. doi:10.1111/j.1528-1167.2009.02487.x

39. Sherman E, Slick DJ, Connolly MB, et al. Reexamining the effects of epilepsy surgery on IQ in children: use of regression-based change scores. *J Int Neuropsychol Soc*. Sep 2003;9(6):879-86. doi:10.1017/s1355617703960085

40. Shurtleff HA, Barry D, Firman T, et al. Impact of epilepsy surgery on development of preschool children: identification of a cohort likely to benefit from early intervention. *J Neurosurg Pediatr*. Oct 2015;16(4):383-92. doi:10.3171/2015.3.Peds14359

41. Sibilia V, Barba C, Metitieri T, et al. Cognitive outcome after epilepsy surgery in children: A controlled longitudinal study. *Epilepsy Behav*. Aug 2017;73:23-30. doi:10.1016/j.yebeh.2017.03.001

42. Silva JR, Sakamoto AC, Thomé Ú, et al. Left hemispherectomy in older children and adolescents: outcome of cognitive abilities. *Childs Nerv Syst*. Jun 2020;36(6):1275-1282. doi:10.1007/s00381-019-04377-9

43. Skirrow C, Cross JH, Cormack F, Harkness W, Vargha-Khadem F, Baldeweg T. Long-term intellectual outcome after temporal lobe surgery in childhood. *Neurology*. Apr 12 2011;76(15):1330-7. doi:10.1212/WNL.0b013e31821527f0

44. Skirrow C, Cross JH, Owens R, et al. Determinants of IQ outcome after focal epilepsy surgery in childhood: A longitudinal case-control neuroimaging study. *Epilepsia*. May 2019;60(5):872-884. doi:10.1111/epi.14707

45. Souza-Oliveira C, Escorsi-Rosset S, Terra VC, Muxfeldt-Bianchin M, Machado HR, Sakamoto AC. Impact of pediatric epilepsy surgery on intellectual efficiency. *Rev Neurol*. Feb 16 2012;54(4):214-20.

46. Sugano H, Nakanishi H, Nakajima M, et al. Posterior quadrant disconnection surgery for Sturge-Weber syndrome. *Epilepsia*. May 2014;55(5):683-689. doi:10.1111/epi.12547

47. Tavares TP, Kerr EN, Smith ML. Memory outcomes following hemispherectomy in children. *Epilepsy Behav*. Nov 2020;112:107360. doi:10.1016/j.yebeh.2020.107360

48. Vadera S, Kshettry VR, Klaas P, Bingaman W. Seizure-free and neuropsychological outcomes after temporal lobectomy with amygdalohippocampectomy in pediatric patients with hippocampal sclerosis. *J Neurosurg Pediatr*. Aug 2012;10(2):103-7. doi:10.3171/2012.4.Peds1233

49. Van Oijen M, De Waal H, Van Rijen PC, Jennekens-Schinkel A, van Huffelen AC, Van Nieuwenhuizen O. Resective epilepsy surgery in childhood: the Dutch experience 1992-2002. *Eur J Paediatr Neurol*. May 2006;10(3):114-23. doi:10.1016/j.ejpn.2006.04.003

50. van Schooneveld MMJ, Jennekens-Schinkel A, van Rijen PC, Braun KPJ, van Nieuwenhuizen O, Dutch Collaborative Epilepsy S. Hemispherectomy: a basis for mental development in children with epilepsy. *Epileptic Disorders*. Mar 2011;13(1):47-55. doi:10.1684/epd.2011.0403

51. Veersema TJ, van Schooneveld MMJ, Ferrier CH, et al. Cognitive functioning after epilepsy surgery in children with mild malformation of cortical development and focal cortical dysplasia. *Epilepsy Behav*. May 2019;94:209-215. doi:10.1016/j.yebeh.2019.03.009

52. Viggedal G, Kristjansdottir R, Olsson I, Rydenhag B, Uvebrant P. Cognitive development from two to ten years after pediatric epilepsy surgery. *Epilepsy Behav*. Sep 2012;25(1):2-8. doi:10.1016/j.yebeh.2012.06.010

53. Villarejo-Ortega F, García-Fernández M, Fournier-Del Castillo C, et al. Seizure and developmental outcomes after hemispherectomy in children and adolescents with intractable epilepsy. *Childs Nerv Syst*. Mar 2013;29(3):475-88. doi:10.1007/s00381-012-1949-8

54. Wang Y, Wang D, Li D, et al. Improvement of intellectual outcomes in 20 children with refractory epilepsy after individualized surgery. *Surg Neurol Int*. 2018;9:203. doi:10.4103/sni.sni_381_17

55. Westerveld M, Sass KJ, Chelune GJ, et al. Temporal lobectomy in children: cognitive outcome. *J Neurosurg*. Jan 2000;92(1):24-30. doi:10.3171/jns.2000.92.1.0024

56. Yang PF, Mei Z, Lin Q, et al. Disconnective surgery in posterior quadrantic epilepsy: a series of 12 paediatric patients. *Epileptic Disord*. Sep 2014;16(3):296-304. doi:10.1684/epd.2014.0678

57. Zaroff CM, Morrison C, Ferraris N, Weiner HL, Miles DK, Devinsky O. Developmental outcome of epilepsy surgery in tuberous sclerosis complex. *Epileptic Disord*. Dec 2005;7(4):321-6.
