# Supplementary material for: IQ changes after pediatric epilepsy surgery: a systematic review and meta-analysis
Source: J Neurol. 2023 Sep 28;271(1):177–87. doi: 10.1007/s00415-023-12002-8 (PMC10770207; doi:10.1007/s00415-023-12002-8)
Supplement: Supplementary file 3 — Supplementary file3 (DOCX 20 KB) [file 415_2023_12002_MOESM3_ESM.docx]

| **Omitted study** | **Mean diff.** | **95% Conf. interval** | **p value** | **I²** |
| --- | --- | --- | --- | --- |
| Ikegaya 2020 | 2.57 | 1.17 – 3.97 | <0.001 | 0% |
| BJØRNÆS 2002 | 2.59 | 1.19 – 3.99 | <0.001 | 0% |
| Silva 2020 | 2.65 | 1.24 – 4.07 | <0.001 | 0% |
| Marashly 2019 | 2.60 | 1.20 – 4.01 | <0.001 | 0% |
| Hallböök 2013 | 2.64 | 1.23 – 4.05 | <0.001 | 0% |
| Arya et al. 2019 | 2.66 | 1.24 – 4.08 | <0.001 | 0% |
| Tavares 2020 | 2.63 | 1.22 – 4.05 | <0.001 | 0% |
| Puka 2015 | 2.88 | 1.42 – 4.34 | <0.001 | 0% |
| Sherman 2003 | 2.57 | 1.16 – 3.98 | <0.001 | 0% |
| Ferguson 2021 | 2.63 | 1.21 – 4.05 | <0.001 | 0% |
| Ramantani 2018 | 2.58 | 1.17 – 4.00 | <0.001 | 0% |
| Souza-Oliveira 2012 | 2.58 | 1.17 – 3.99 | <0.001 | 0% |
| Kuehn 2002 | 2.55 | 1.14 – 3.96 | <0.001 | 0% |
| Barba et al. 2020 | 2.58 | 1.13 – 4.03 | <0.001 | 0% |
| Ko 2019 | 2.52 | 1.12 – 3.93 | <0.001 | 0% |
| Battaglia 2012 | 2.53 | 1.12 – 3.94 | <0.001 | 0% |
| Vadera 2012 | 2.53 | 1.11 – 3.94 | <0.001 | 0% |
| Miranda 2001 | 2.52 | 1.09 – 3.95 | <0.001 | 0% |
| Viggedal 2012 | 2.52 | 1.12 – 3.91 | <0.001 | 0% |
| Laguitton 2021 | 2.51 | 1.07 – 3.95 | <0.001 | 0% |
| Garcia-Fernandez 2011 | 2.51 | 1.11 – 3.92 | <0.001 | 0% |
| Van Oijen 2006 | 2.52 | 1.12 – 3.91 | <0.001 | 0% |
| Van Schooneveld 2011 | 2.49 | 1.08 – 3.91 | <0.001 | 0% |
| Benova 2019 | 2.40 | 0.93 – 3.86 | <0.001 | 0% |
| Chandra 2018 | 2.47 | 1.05 – 3.89 | <0.001 | 0% |
| Veersema 2019 | 2.47 | 1.05 – 3.89 | <0.001 | 0% |
| Ramantani 2014 | 2.50 | 1.10 – 3.90 | <0.001 | 0% |
| Wang 2018 | 2.51 | 1.11 – 3.91 | <0.001 | 0% |
| Yang 2014 | 2.48 | 1.08 – 3.89 | <0.001 | 0% |
| Jayalakshimi 2014 | 2.50 | 1.10 – 3.90 | <0.001 | 0% |
| Skirrow 2019 | 2.43 | 1.01 – 3.84 | <0.001 | 0% |
| Freitag 2005 | 2.49 | 1.09 – 3.89 | <0.001 | 0% |
| Qu 2020 | 2.40 | 0.99 – 3.82 | <0.001 | 0% |
| Elliot 2000 | 2.48 | 1.08 – 3.88 | <0.001 | 0% |
| Skirrow 2011 | 2.48 | 1.08 – 3.88 | <0.001 | 0% |
| Shurtleff 2015 | 2.47 | 1.07 – 3.87 | <0.001 | 0% |
| Liu 2007 | 2.08 | 0.65 – 3.50 | 0.004 | 0% |
| Liu 2012 | 2.14 | 0.72 – 3.55 | 0.003 | 0% |
| **Total** | **2.52** | **1.12 – 3.91** | **<0.001** | **0%** |

# S3: Leave one out analysis

# S3.1: Mean change of FSIQ

# S3.2: ASM free vs. Not ASM free

| **Omitted Study** | **Mean difference** | **95% Conf. interval** | **P - value** | **I²** |
| --- | --- | --- | --- | --- |
| Battaglia 2012 | 4.94 | 2.43 – 7.44 | <0.001 | 0% |
| Marashly 2019 | 4.39 | 2.07 – 6.70 | <0.001 | 0% |
| Jayalakshimi 2014 | 4.49 | 1.13 – 7.85 | 0.009 | 0% |
| Viggedal 2012 | 4.35 | 2.13 – 6.57 | <0.001 | 0% |
| Shurtleff 2015 | 4.24 | 2.01 – 6.48 | <0.001 | 0% |
| Ramantani 2014 | 3.88 | 1.58 – 6.19 | <0.001 | 0% |
| **Total** | **4.35** | **2.15 – 6.56** | **<0.001** | **0%** |

# S3.3: Seizure free vs. Not seizure free

| **Omitted Study** | **Mean difference** | **95% Conf. interval** | **P - value** | **I²** |
| --- | --- | --- | --- | --- |
| Ikegaya 2020 | 6.05 | 2.11 – 10.00 | 0.003 | 76% |
| Van Schooneveld 2011 | 5.69 | 1.69 – 9.70 | 0.005 | 78% |
| Sherman 2003 | 5.87 | 1.81 – 9.93 | 0.005 | 77% |
| Marashly 2019 | 5.88 | 1.81 – 9.95 | 0.005 | 77% |
| Benova 2019 | 5.79 | 1.71 – 9.88 | 0.005 | 78% |
| Ramantani 2014 | 5.61 | 1.53 – 9.69 | 0.007 | 78% |
| Jayalakshimi 2014 | 5.49 | 1.03 – 9.96 | 0.02 | 78% |
| Battaglia 2012 | 5.14 | 0.57 – 9.70 | 0.03 | 79% |
| Liu 2007 | 5.08 | 0.74 – 9.42 | 0.02 | 79% |
| Liu 2012 | 4.86 | 0.85 – 8.88 | 0.02 | 78% |
| Freitag 2005 | 4.62 | 0.66 – 8.58 | 0.02 | 77% |
| Qu 2020 | 3.91 | 1.12 – 6.70 | 0.006 | 47% |
| **Total** | **5.34** | **1.47 – 9.21** | **0.007** | **77%** |
