# Supplementary figures and images for: IQ changes after pediatric epilepsy surgery: a systematic review and meta-analysis
Source: J Neurol. 2023 Sep 28;271(1):177–87. doi: 10.1007/s00415-023-12002-8 (PMC10770207; doi:10.1007/s00415-023-12002-8)

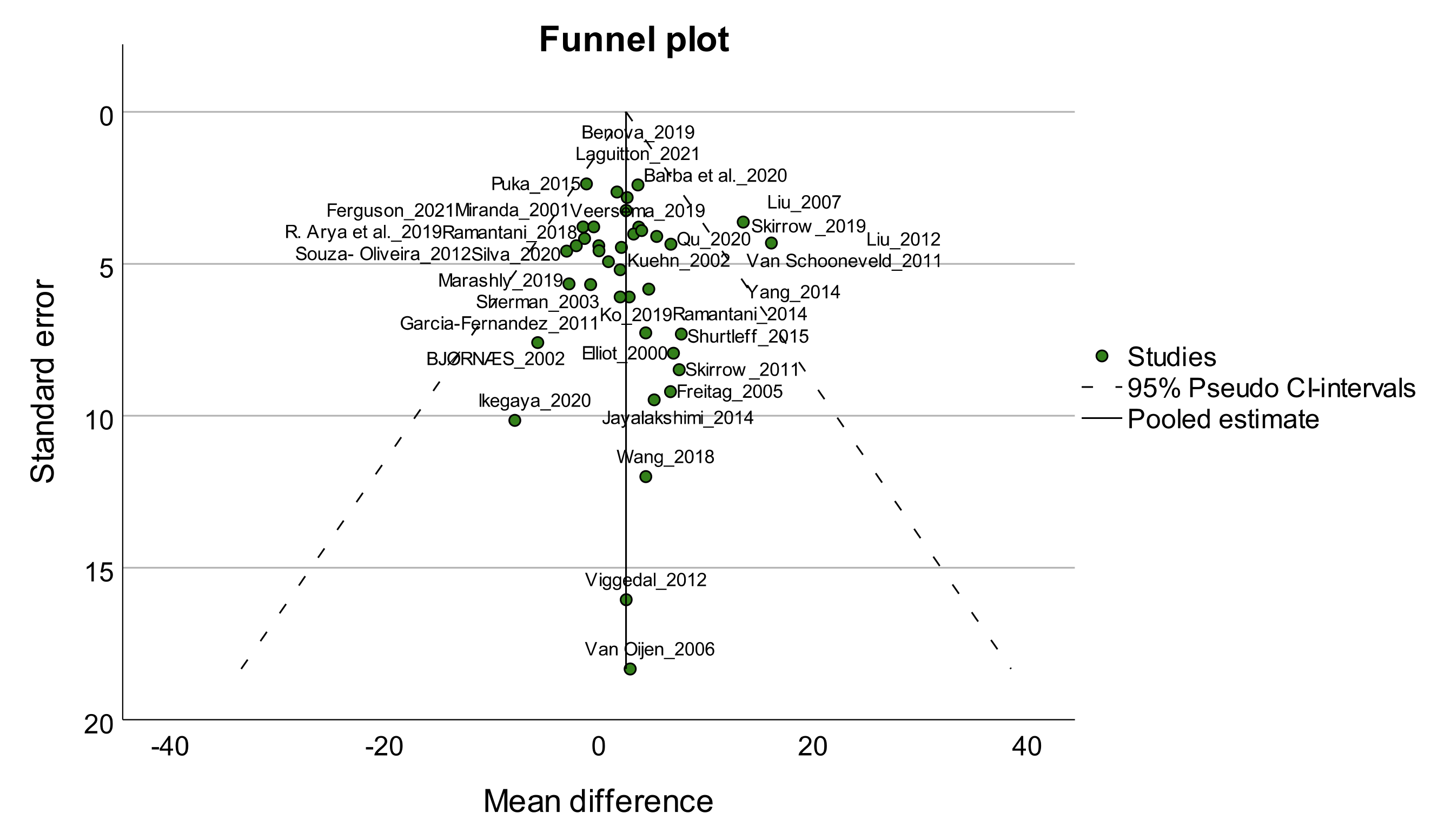

Supplement: Supplementary file 4 — Online Resource 4: Funnel plot reveals evenly distributed studies, indicating a low risk of bias (TIFF 16063 KB) [file 415_2023_12002_MOESM4_ESM.tiff]
